# Supplementary material for: A Likelihood Approach for Real-Time Calibration of Stochastic Compartmental Epidemic Models
Source: PLoS Comput Biol. 2017 Jan 17;13(1):e1005257. doi: 10.1371/journal.pcbi.1005257 (PMC5240920; doi:10.1371/journal.pcbi.1005257)
Supplement: S1 File — (TAR.GZ) [file pcbi.1005257.s014.tar.gz › HSPH_Online-SI-Revision/Additions/IPoi10/n100-extreme-SEITR_noisy/n100-extreme-SEITR_noisy_BENCH_table8.pdf]

1	R0	{1.38654, 9.85565}	{2.86095, 19.9929}	{{2.86095, 19.9929}, {2.86095, 19.9929}}	{106.337, 102.857}	inf	78.	6648.08	{3377., 10 122.}	910.346	0.96109	newly	368.386	2047.17	{1031., 3105.}	455.714	0.70073	Reff	0.108483	0.258405	{0.225328, 0.293934}	138.199	ar	92 379.	93 024.2	{92 875., 93 205.}	0.698464
2	R0	{2.36629, 12.2029}	{2.9884, 12.8326}	{{2.9884, 12.8326}, {2.9884, 12.8326}}	{26.2905, 5.16025}	inf	5.	2873.77	{1311., 5430.}	317.092	0.541245	newly	300.	1265.67	{575., 2452.}	321.891	0.545649	Reff	0.0235682	0.201785	{0.189046, 0.223532}	756.172	ar	99 022.	93 951.9	{93 830., 94 122.}	5.1202
3	R0	{2.32253, 12.836}	{2.9194, 10.1535}	{{2.9194, 10.1535}, {2.9194, 10.1535}}	{25.6991, 20.8983}	inf	20.	569.085	{260., 982.}	52.6811	0.357752	newly	463.	300.507	{139., 505.}	47.1664	0.291843	Reff	0.0245491	0.194823	{0.189732, 0.200388}	693.603	ar	98 997.	93 465.	{93 320., 93 628.}	5.58805
4	R0	{1.79075, 11.2058}	{2.9884, 12.8326}	{{2.9884, 12.8326}, {2.9884, 12.8326}}	{66.8798, 14.5175}	inf	31.	1903.86	{760., 3725.}	133.33	0.305361	newly	412.	842.764	{342., 1651.}	110.818	0.269147	Reff	0.0574114	0.193238	{0.184115, 0.209427}	236.584	ar	96 920.	93 982.6	{93 861., 94 122.}	3.03076
5	R0	{2.26952, 9.62371}	{2.98388, 9.60029}	{{2.98388, 9.60029}, {2.98388, 9.60029}}	{31.4763, 0.243357}	inf	0.	489.623	{266., 866.}	162.041	0.371793	newly	78.	268.148	{147., 474.}	243.779	0.488841	Reff	0.0257137	0.184681	{0.180555, 0.18885}	618.22	ar	98 881.	93 921.5	{93 795., 94 030.}	5.01563
6	R0	{1.57191, 6.89004}	{2.9194, 10.1535}	{{2.9194, 10.1535}, {2.9194, 10.1535}}	{85.7231, 47.3649}	inf	5.	615.385	{344., 1042.}	1039.6	0.997346	newly	40.	325.631	{182., 561.}	714.077	0.850755	Reff	0.077951	0.195154	{0.190724, 0.200183}	150.354	ar	95 046.	93 464.	{93 334., 93 598.}	1.66441
7	R0	{1.99226, 17.2339}	{2.97157, 19.3547}	{{2.97157, 19.3547}, {2.97157, 19.3547}}	{49.1557, 12.306}	inf	78.	4315.75	{2131., 7871.}	66.4932	0.189565	newly	982.	1369.97	{670., 2548.}	60.7211	0.184725	Reff	0.0456427	0.216149	{0.195292, 0.2519}	373.568	ar	97 992.	93 859.4	{93 725., 94 004.}	4.21729
8	R0	{2.63369, 15.1879}	{2.9884, 12.8326}	{{2.9884, 12.8326}, {2.9884, 12.8326}}	{13.4682, 15.5077}	inf	5.	2305.94	{1277., 3698.}	66.9489	0.195335	newly	510.	1018.25	{546., 1650.}	101.158	0.266905	Reff	0.0154861	0.196149	{0.18818, 0.207395}	1166.61	ar	99 451.	93 972.1	{93 836., 94 099.}	5.50911
9	R0	{2.53271, 9.90552}	{2.98388, 9.60029}	{{2.98388, 9.60029}, {2.98388, 9.60029}}	{17.8137, 3.08141}	inf	2.	470.333	{242., 813.}	175.42	0.38606	newly	96.	257.746	{130., 447.}	169.279	0.376481	Reff	0.0167159	0.1838	{0.178824, 0.189805}	999.555	ar	99 355.	93 944.9	{93 799., 94 101.}	5.44522
10	R0	{2.48791, 18.9003}	{2.95376, 15.278}	{{2.95376, 15.278}, {2.95376, 15.278}}	{18.7246, 19.1653}	inf	42.	2954.57	{1232., 6284.}	51.3053	0.285008	newly	1167.	1136.91	{458., 2380.}	54.074	0.248793	Reff	0.0212716	0.206684	{0.192142, 0.231811}	871.644	ar	99 291.	93 747.4	{93 592., 93 914.}	5.58314
11	R0	{2.84414, 18.4765}	{2.86118, 15.5722}	{{2.86118, 15.5722}, {2.86118, 15.5722}}	{0.599127, 15.7189}	inf	13.	3841.7	{1855., 6697.}	53.3514	0.191228	newly	1003.	1458.75	{650., 2561.}	65.096	0.197466	Reff	0.0117463	0.231316	{0.211727, 0.258136}	1869.27	ar	99 654.	93 008.8	{92 864., 93 150.}	6.6683
12	R0	{2.29369, 14.622}	{2.7943, 13.5962}	{{2.7943, 13.5962}, {2.7943, 13.5962}}	{21.8255, 7.01546}	inf	14.	3271.84	{1203., 7765.}	145.405	0.291824	newly	542.	1400.78	{511., 3312.}	162.527	0.313531	Reff	0.0247948	0.239553	{0.219995, 0.280408}	866.144	ar	98 982.	92 445.3	{92 329., 92 604.}	6.60389
13	R0	{2.73413, 16.7529}	{2.99939, 13.7076}	{{2.99939, 13.7076}, {2.99939, 13.7076}}	{9.70181, 18.1777}	inf	10.	1761.33	{916., 3003.}	37.6859	0.189726	newly	706.	737.192	{370., 1284.}	38.0011	0.159887	Reff	0.0138347	0.190777	{0.182063, 0.201169}	1278.97	ar	99 543.	94 041.9	{93 885., 94 184.}	5.52634
14	R0	{1.79554, 16.5943}	{2.76795, 19.6041}	{{2.76795, 19.6041}, {2.76795, 19.6041}}	{54.157, 18.1376}	inf	185.	4621.43	{2336., 8304.}	41.9595	0.151828	newly	1313.	1453.22	{705., 2673.}	37.3335	0.148602	Reff	0.0662734	0.25665	{0.23511, 0.290718}	287.259	ar	96 821.	92 199.4	{92 038., 92 352.}	4.77337
15	R0	{1.91429, 13.7771}	{2.97157, 19.3547}	{{2.97157, 19.3547}, {2.97157, 19.3547}}	{55.2309, 40.4846}	inf	37.	4533.88	{1885., 6890.}	208.756	0.401923	newly	621.	1426.97	{588., 2206.}	132.944	0.286583	Reff	0.0501544	0.21937	{0.194281, 0.235111}	337.389	ar	97 541.	93 875.2	{93 732., 93 992.}	3.75821
16	R0	{1.42094, 10.5871}	{2.8939, 16.8013}	{{2.8939, 16.8013}, {2.8939, 16.8013}}	{103.661, 58.696}	inf	126.	3260.57	{1521., 6371.}	171.488	0.37565	newly	644.	1159.35	{540., 2285.}	85.8651	0.224798	Reff	0.103388	0.220645	{0.206393, 0.247081}	113.416	ar	93 044.	93 258.9	{93 132., 93 381.}	0.232179
17	R0	{1.45515, 6.16466}	{2.9194, 10.1535}	{{2.9194, 10.1535}, {2.9194, 10.1535}}	{100.625, 64.7049}	inf	5.	1199.42	{556., 2402.}	2298.85	1.30619	newly	41.	636.333	{294., 1258.}	1452.03	1.11683	Reff	0.0982954	0.199407	{0.191921, 0.210693}	102.865	ar	93 254.	93 460.8	{93 311., 93 601.}	0.226442
18	R0	{2.85669, 13.2416}	{2.83858, 10.6973}	{{2.83858, 10.6973}, {2.83858, 10.6973}}	{0.63395, 19.2144}	inf	1.	1236.86	{596., 2207.}	69.8976	0.195187	newly	314.	632.803	{296., 1114.}	105.216	0.259271	Reff	0.0106269	0.213073	{0.206507, 0.221154}	1905.04	ar	99 644.	92 823.7	{92 671., 92 991.}	6.84462
19	R0	{2.56417, 17.9558}	{2.8939, 16.8013}	{{2.8939, 16.8013}, {2.8939, 16.8013}}	{12.8591, 6.42968}	inf	35.	6072.69	{2944., 10 157.}	94.2004	0.232428	newly	1136.	2206.75	{1047., 3624.}	98.4118	0.234696	Reff	0.01818	0.263406	{0.216579, 0.291995}	1348.88	ar	99 389.	93 234.8	{93 061., 93 413.}	6.19203
20	R0	{2.27517, 11.1864}	{2.92375, 10.9639}	{{2.92375, 10.9639}, {2.92375, 10.9639}}	{28.5069, 1.98902}	inf	9.	1124.23	{542., 1894.}	144.753	0.344861	newly	219.	563.231	{285., 934.}	157.633	0.367857	Reff	0.0255502	0.197569	{0.190745, 0.204604}	673.261	ar	98 894.	93 515.8	{93 360., 93 663.}	5.43839
21	R0	{2.35735, 10.4735}	{2.9194, 10.1535}	{{2.9194, 10.1535}, {2.9194, 10.1535}}	{23.8425, 3.05533}	inf	3.	1145.15	{543., 1799.}	282.992	0.507204	newly	156.	604.647	{292., 941.}	287.594	0.511678	Reff	0.0230549	0.198092	{0.190841, 0.205263}	759.219	ar	99 036.	93 502.	{93 375., 93 650.}	5.58784
22	R0	{1.79551, 11.9179}	{2.7943, 13.5962}	{{2.7943, 13.5962}, {2.7943, 13.5962}}	{55.6271, 14.0822}	inf	31.	1391.02	{665., 2018.}	68.796	0.182817	newly	400.	590.758	{279., 878.}	61.8347	0.169599	Reff	0.0555172	0.221898	{0.214211, 0.22849}	299.693	ar	97 033.	92 453.8	{92 300., 92 599.}	4.7192
23	R0	{1.23545, 6.31687}	{2.9884, 12.8326}	{{2.9884, 12.8326}, {2.9884, 12.8326}}	{141.888, 103.148}	inf	19.	1746.03	{770., 3023.}	1663.66	1.16942	newly	79.	775.442	{342., 1360.}	881.572	0.914036	Reff	0.139655	0.193412	{0.184235, 0.20608}	38.4927	ar	88 739.	93 936.8	{93 784., 94 086.}	5.85745
24	R0	{2.24089, 10.1789}	{2.83858, 10.6973}	{{2.83858, 10.6973}, {2.83858, 10.6973}}	{26.672, 5.09289}	inf	7.	1308.75	{553., 2310.}	277.16	0.498865	newly	171.	673.194	{278., 1163.}	293.681	0.516802	Reff	0.025703	0.213781	{0.2054, 0.225213}	731.736	ar	98 868.	92 816.8	{92 656., 92 963.}	6.12046
25	R0	{1.57298, 10.2607}	{2.86118, 15.5722}	{{2.86118, 15.5722}, {2.86118, 15.5722}}	{81.8955, 51.7655}	inf	47.	5129.88	{2373., 9367.}	489.642	0.710298	newly	443.	1964.6	{919., 3637.}	343.477	0.585757	Reff	0.0819837	0.243392	{0.215733, 0.283743}	196.878	ar	94 957.	93 014.9	{92 855., 93 166.}	2.0452
26	R0	{1.2154, 8.2576}	{2.8939, 16.8013}	{{2.8939, 16.8013}, {2.8939, 16.8013}}	{138.103, 103.465}	inf	88.	4265.49	{2387., 6908.}	712.475	0.86115	newly	345.	1527.89	{822., 2406.}	342.866	0.596636	Reff	0.143952	0.229529	{0.210444, 0.255445}	59.4481	ar	88 421.	93 259.7	{93 091., 93 418.}	5.47233
27	R0	{2.22841, 18.7983}	{2.8833, 17.1847}	{{2.8833, 17.1847}, {2.8833, 17.1847}}	{29.3882, 8.58375}	inf	67.	2503.28	{1348., 4238.}	38.8291	0.221163	newly	1079.	874.284	{469., 1455.}	35.6328	0.187313	Reff	0.0322005	0.215342	{0.203763, 0.234268}	568.755	ar	98 756.	93 188.3	{93 036., 93 347.}	5.63781
28	R0	{1.27778, 5.36767}	{2.98388, 9.60029}	{{2.98388, 9.60029}, {2.98388, 9.60029}}	{133.521, 78.854}	inf	7.	256.537	{128., 477.}	654.522	0.817721	newly	32.	141.746	{66., 254.}	342.957	0.582331	Reff	0.130065	0.182413	{0.178496, 0.186821}	40.2474	ar	89 843.	93 943.6	{93 807., 94 082.}	4.5642
29	R0	{2.38042, 15.8901}	{2.99939, 13.7076}	{{2.99939, 13.7076}, {2.99939, 13.7076}}	{26.0026, 13.735}	inf	37.	1171.2	{600., 1711.}	55.373	0.367812	newly	849.	488.031	{234., 691.}	51.0969	0.319851	Reff	0.0237804	0.186646	{0.180083, 0.192441}	684.875	ar	99 112.	94 040.4	{93 887., 94 218.}	5.11706
30	R0	{2.53188, 12.7607}	{2.9194, 10.1535}	{{2.9194, 10.1535}, {2.9194, 10.1535}}	{15.3056, 20.4315}	inf	11.	1139.58	{532., 2263.}	49.8103	0.183806	newly	428.	605.61	{288., 1213.}	62.7989	0.185768	Reff	0.0182295	0.198774	{0.190812, 0.206664}	990.393	ar	99 317.	93 470.7	{93 307., 93 631.}	5.88653
31	R0	{1.24482, 10.5471}	{2.86095, 19.9929}	{{2.86095, 19.9929}, {2.86095, 19.9929}}	{129.828, 89.5583}	inf	170.	5430.74	{2478., 9564.}	324.277	0.556478	newly	689.	1677.97	{756., 2905.}	144.656	0.317557	Reff	0.142009	0.249248	{0.218577, 0.286639}	75.5157	ar	89 097.	92 991.7	{92 852., 93 135.}	4.37135
32	R0	{2.30446, 17.9607}	{2.7943, 13.5962}	{{2.7943, 13.5962}, {2.7943, 13.5962}}	{21.2562, 24.3003}	inf	48.	2806.57	{894., 3925.}	71.1206	0.344104	newly	1094.	1293.06	{382., 1696.}	83.2161	0.277269	Reff	0.0266626	0.269711	{0.218905, 0.249978}	911.572	ar	99 028.	92 413.	{92 261., 92 559.}	6.67994
33	R0	{2.31579, 11.8236}	{2.98388, 9.60029}	{{2.98388, 9.60029}, {2.98388, 9.60029}}	{28.8493, 18.804}	inf	5.	129.159	{63., 231.}	72.9226	0.623231	newly	197.	71.0435	{34., 130.}	64.7024	0.500866	Reff	0.0234821	0.181922	{0.177571, 0.186463}	674.728	ar	99 012.	93 931.7	{93 783., 94 074.}	5.13095
34	R0	{1.6678, 10.6744}	{2.96307, 14.3219}	{{2.96307, 14.3219}, {2.96307, 14.3219}}	{77.6634, 34.1705}	inf	36.	2247.15	{920., 5225.}	292.892	0.508619	newly	278.	911.717	{356., 2090.}	228.444	0.432121	Reff	0.0712985	0.19966	{0.186999, 0.221697}	180.034	ar	95 809.	93 813.8	{93 684., 93 953.}	2.08243
35	R0	{1.65476, 7.07559}	{2.98388, 9.60029}	{{2.98388, 9.60029}, {2.98388, 9.60029}}	{80.321, 35.6818}	inf	5.	555.912	{216., 827.}	705.669	0.792587	newly	50.	305.853	{116., 457.}	511.706	0.672059	Reff	0.0705259	0.184982	{0.179152, 0.189476}	162.289	ar	95 746.	93 928.6	{93 784., 94 079.}	1.89813
36	R0	{2.71807, 18.5959}	{2.95376, 15.278}	{{2.95376, 15.278}, {2.95376, 15.278}}	{8.67123, 17.8421}	inf	24.	4437.12	{2481., 8741.}	44.2555	0.150407	newly	1211.	1707.06	{966., 3366.}	52.8144	0.149026	Reff	0.0154386	0.220668	{0.201889, 0.255677}	1329.33	ar	99 540.	93 719.3	{93 553., 93 900.}	5.84757
37	R0	{2.6202, 12.7552}	{2.99939, 13.7076}	{{2.99939, 13.7076}, {2.99939, 13.7076}}	{14.4718, 7.46676}	inf	9.	2562.42	{1260., 4624.}	193.519	0.404817	newly	383.	1076.32	{554., 1932.}	181.023	0.385388	Reff	0.016088	0.196797	{0.184762, 0.216076}	1123.25	ar	99 426.	94 045.8	{93 902., 94 169.}	5.41122
38	R0	{1.47672, 9.81651}	{2.86118, 15.5722}	{{2.86118, 15.5722}, {2.86118, 15.5722}}	{93.7524, 58.6328}	inf	40.	2361.99	{1080., 4330.}	315.112	0.556838	newly	323.	892.493	{384., 1700.}	176.61	0.380098	Reff	0.0934468	0.217605	{0.205404, 0.233644}	132.865	ar	93 815.	93 032.8	{92 903., 93 184.}	0.833756
39	R0	{1.90004, 12.3313}	{2.7943, 13.5962}	{{2.7943, 13.5962}, {2.7943, 13.5962}}	{47.0653, 10.2576}	inf	30.	1932.65	{956., 3056.}	86.4307	0.224877	newly	464.	821.413	{412., 1277.}	81.5921	0.214864	Reff	0.0517191	0.226232	{0.216446, 0.235308}	337.424	ar	97 413.	92 449.7	{92 273., 92 611.}	5.09506
40	R0	{2.64218, 18.5237}	{2.99939, 13.7076}	{{2.99939, 13.7076}, {2.99939, 13.7076}}	{13.5195, 25.9997}	inf	25.	1609.2	{576., 3158.}	58.1465	0.385937	newly	874.	672.606	{244., 1310.}	54.9376	0.303307	Reff	0.0162494	0.190165	{0.180863, 0.204648}	1070.29	ar	99 474.	94 032.7	{93 860., 94 176.}	5.47004
41	R0	{1.6945, 7.17996}	{2.9194, 10.1535}	{{2.9194, 10.1535}, {2.9194, 10.1535}}	{72.2868, 41.4144}	inf	4.	1068.16	{460., 1963.}	937.044	0.929689	newly	74.	564.	{233., 1016.}	662.162	0.793441	Reff	0.0626626	0.198232	{0.19198, 0.207482}	216.349	ar	96 332.	93 473.2	{93 348., 93 608.}	2.96765
42	R0	{1.84559, 18.0074}	{2.93978, 19.9817}	{{2.93978, 19.9817}, {2.93978, 19.9817}}	{59.2867, 10.9638}	inf	180.	5555.41	{2882., 8777.}	52.7618	0.154056	newly	1374.	1715.15	{879., 2664.}	45.7501	0.146632	Reff	0.0586713	0.233782	{0.208842, 0.257966}	298.461	ar	97 358.	93 634.5	{93 467., 93 789.}	3.82456
43	R0	{2.24965, 17.0361}	{2.95376, 15.278}	{{2.95376, 15.278}, {2.95376, 15.278}}	{31.2986, 10.3199}	inf	59.	2482.43	{1212., 4229.}	39.7848	0.212787	newly	1018.	952.174	{469., 1617.}	37.9915	0.177957	Reff	0.0319	0.202651	{0.19037, 0.215802}	535.268	ar	98 770.	93 743.7	{93 577., 93 903.}	5.08891
44	R0	{2.54324, 15.7967}	{2.7943, 13.5962}	{{2.7943, 13.5962}, {2.7943, 13.5962}}	{9.87166, 13.9301}	inf	13.	2552.71	{1337., 4040.}	65.5424	0.192942	newly	626.	1087.45	{563., 1724.}	78.5313	0.217727	Reff	0.0177772	0.231998	{0.218458, 0.248385}	1205.03	ar	99 354.	92 439.3	{92 251., 92 628.}	6.95966
45	R0	{2.54943, 14.8032}	{2.9884, 12.8326}	{{2.9884, 12.8326}, {2.9884, 12.8326}}	{17.2184, 13.312}	inf	8.	2556.75	{1204., 4748.}	93.2608	0.236854	newly	536.	1136.51	{548., 2119.}	115.29	0.276294	Reff	0.0170047	0.19805	{0.186177, 0.216031}	1064.68	ar	99 380.	93 977.2	{93 816., 94 121.}	5.43648
46	R0	{2.37755, 12.6461}	{2.9194, 10.1535}	{{2.9194, 10.1535}, {2.9194, 10.1535}}	{22.7903, 19.7104}	inf	9.	428.614	{156., 611.}	73.9894	0.521062	newly	377.	226.471	{84., 326.}	71.2202	0.440874	Reff	0.0224678	0.193078	{0.187513, 0.197965}	759.352	ar	99 096.	93 491.8	{93 321., 93 645.}	5.65531
47	R0	{1.69279, 11.6636}	{2.95376, 15.278}	{{2.95376, 15.278}, {2.95376, 15.278}}	{74.4906, 30.9887}	inf	61.	2332.4	{1150., 3852.}	101.699	0.25861	newly	550.	893.114	{453., 1451.}	71.3039	0.200955	Reff	0.0676608	0.202908	{0.192526, 0.216097}	199.89	ar	96 214.	93 699.5	{93 589., 93 832.}	2.61343
48	R0	{1.82402, 10.0038}	{2.9884, 12.8326}	{{2.9884, 12.8326}, {2.9884, 12.8326}}	{63.8359, 28.2773}	inf	27.	1791.1	{840., 2863.}	263.306	0.506614	newly	248.	792.516	{374., 1267.}	219.563	0.451066	Reff	0.054447	0.192415	{0.18292, 0.201717}	253.399	ar	97 076.	93 973.3	{93 840., 94 102.}	3.19615
49	R0	{1.6424, 11.1667}	{2.99939, 13.7076}	{{2.99939, 13.7076}, {2.99939, 13.7076}}	{82.6224, 22.7543}	inf	29.	824.066	{380., 1526.}	50.1194	0.170504	newly	321.	345.026	{163., 607.}	46.7044	0.183473	Reff	0.0752383	0.184697	{0.178434, 0.191391}	145.482	ar	95 546.	94 028.3	{93 866., 94 191.}	1.58847
50	R0	{2.05638, 14.3048}	{2.99939, 13.7076}	{{2.99939, 13.7076}, {2.99939, 13.7076}}	{45.8578, 4.17482}	inf	38.	1997.74	{876., 3167.}	57.8953	0.18872	newly	636.	839.29	{354., 1315.}	61.5008	0.1906	Reff	0.0397498	0.193287	{0.182513, 0.202219}	386.258	ar	98 194.	94 036.	{93 913., 94 182.}	4.23452
